# Mechanisms of Copper Ion Mediated Huntington's Disease Progression

**DOI:** 10.1371/journal.pone.0000334

**Published:** 2007-03-28

**Authors:** Jonathan H. Fox, Jibrin A. Kama, Gregory Lieberman, Raman Chopra, Kate Dorsey, Vanita Chopra, Irene Volitakis, Robert A. Cherny, Ashley I. Bush, Steven Hersch

**Affiliations:** 1 Department of Neurology, Harvard Medical School, Massachusetts General Hospital, Charlestown, Massachusetts, United States of America; 2 Genetics and Aging Research Unit, and Department of Psychiatry, Harvard Medical School, Massachusetts General Hospital, Charlestown, Massachusetts, United States of America; 3 Oxidation Disorders Laboratory, Mental Health Research Institute of Victoria, and Department of Pathology, The University of Melbourne, Parkville, Victoria, Australia; DER Neurogenetics, National Institute of Neurological Disorders and Stroke, United States of America

## Abstract

Huntington's disease (HD) is caused by a dominant polyglutamine expansion within the N-terminus of huntingtin protein and results in oxidative stress, energetic insufficiency and striatal degeneration. Copper and iron are increased in the striata of HD patients, but the role of these metals in HD pathogenesis is unknown. We found, using inductively-coupled-plasma mass spectroscopy, that elevations of copper and iron found in human HD brain are reiterated in the brains of affected HD transgenic mice. Increased brain copper correlated with decreased levels of the copper export protein, amyloid precursor protein. We hypothesized that increased amounts of copper bound to low affinity sites could contribute to pro-oxidant activities and neurodegeneration. We focused on two proteins: huntingtin, because of its centrality to HD, and lactate dehydrogenase (LDH), because of its documented sensitivity to copper, necessity for normoxic brain energy metabolism and evidence for altered lactate metabolism in HD brain. The first 171 amino acids of wild-type huntingtin, and its glutamine expanded mutant form, interacted with copper, but not iron. N171 reduced Cu^2+^ *in vitro* in a 1∶1 copper∶protein stoichiometry indicating that this fragment is very redox active. Further, copper promoted and metal chelation inhibited aggregation of cell-free huntingtin. We found decreased LDH activity, but not protein, and increased lactate levels in HD transgenic mouse brain. The LDH inhibitor oxamate resulted in neurodegeneration when delivered intra-striatially to healthy mice, indicating that LDH inhibition is relevant to neurodegeneration in HD. Our findings support a role of pro-oxidant copper-protein interactions in HD progression and offer a novel target for pharmacotherapeutics.

## Introduction

Huntington's disease (HD) is a neurodegenerative disorder characterized by progressive motor, cognitive and psychiatric deterioration. HD is caused by a dominant glutamine expansion within the N-terminus of the huntingtin protein that initiates events leading to neuronal loss primarily within the striatum and cerebral cortex. Full-length huntingtin is large (∼350 kD), but it is the smaller N-terminal fragments that are the main mediators of disease progression [1]. These fragments have aberrant interactions with themselves and other biomolecules that result in hallmarks of HD including aggregates [2], transcriptional repression [3], oxidative injury [4] and mitochondrial dysfunction [5].

Copper and iron concentrations are increased in the striata of post-mortem human HD brains [6]. Further, ferritin-iron levels are increased in striata of early clinical HD patients as measured by MRI [7]. However, the role these metals may have in HD is unknown. The basal ganglia are sensitive to increased copper and iron levels. Patients with Wilson's disease (WD) have large increases in liver copper. However, 40% of WD cases present with neurologic symptoms related to copper-mediated striatal degeneration [8]. Patients with ferritin mutations (neuroferritinopathy) accumulate iron and have degeneration in the globus pallidus [9]. These data suggest possible mechanistic overlap between HD and disorders of primary basal ganglia copper and iron accumulation. However, why copper and iron levels increase in HD and the specific pathways through which they may potentiate neurodegeneration are unknown.

Accumulation of copper in HD brain could result in interaction with low affinity binding sites on diverse biomolecules. Aberrant copper-protein interactions have been implicated in the pathogenesis of Alzheimer's, Lou Gehrig's and Parkinson's diseases [10]–[12]. For polyglutamine diseases, including HD, such interactions are not reported. However, copper could promote altered mutant huntingtin conformation, aggregation and/or redox-activity. This would be analogous to the interaction of beta-amyloid with copper, which induces beta-amyloid oligomerization thought to contribute to Alzheimer's disease pathogenesis [10], [13].

Several glycolytic enzymes [14], [15] and mitochondrial dehydrogenases including lactate dehydrogenase (LDH) [15] and succinate dehydrogenase (SDH) [16], [17] are sensitive to copper-mediated inactivation. LDH is a critical component of the astrocyte-neuron lactate shuttle whereby lactate released by astrocytes is used as an energy substrate by neurons [18]. Lactate levels are increased in human HD striata as measured by magnetic resonance spectroscopy [19]. Further, a recent study has shown that pre-symptomatic N171-82Q HD mice have impaired ability to metabolize lactate, but not succinate [20]. Taken together, these findings suggest that excess copper could contribute to HD energetic insufficiency by inhibiting key enzymes such as LDH.

The major goal of this study was to address the hypothesis that aberrant copper-protein interactions contribute to HD progression. We specifically addressed two potential ways in which copper could contribute to HD pathogenesis, 1.) by modulation of huntingtin structure, and 2.) by interfering with brain lactate-energy metabolism, by inhibiting LDH activity. Our findings indicate that copper could be a hereto unappreciated potentiating factor in HD that may impact pathogenesis at the level of mutant huntingtin itself as well as interfering with brain energy metabolism.

## Materials and Methods

### Materials

All reagents were from Sigma unless otherwise stated. Antibodies used were anti-APLP2 (Calbiochem), anti-ATP7A (Abcam), anti-ATP7B N-terminus antibody (Novus), anti-APP (MAB348 from Chemicon; 6E10 from Signet), 1C2 (anti-polyglutamine) (Chemicon) and anti-SOD1 (Chemicon). Dr. Marian DiFiglia, and Dr. Wilma Wasco kindly provided AB1 (detects first 17 amino acids of huntingtin) and APLP1 antibodies, respectively. HRP-conjugated secondary antibodies were from Abcam.

### N-terminal huntingtin synthesis

A wheat-germ T7-based *in-vitro* transcription-translation system was used to generate N-terminal huntingin (Promega). Constructs used comprised pcDNA1 and expressed the first 171 amino acids of human huntingtin (N171) containing 17 glutamines, or 68 glutamines. Histidine mutants were generated using QuikChange® site-directed mutagenesis kit (Stratagene). To generate purified N171 huntingtin the above vector inserts were sub-cloned into BamH1/Not1 sites of pGEX-6P (Amersham). Constructs were transformed into E.coli strain BL21. Bacteria were grown to OD600 0.5 at 37°C. Cultures were then treated with 1 mM IPTG for 3 hours at 37 or 25°C, for wild-type and mutant protein, respectively. N-171 huntingtin fragments were purified using a GST affinity column and then the GST tag cleaved using PreScission Protease (Amersham). The exon-1-17Q construct was generated by introducing a stop codon into the N171 construct using QuikChange®. For metal reduction assays the N-171-17Q protein was further purified using a Zebra™ desalt spin column (Pierce) then concentrated to ∼300 ng/µl using a 9 kDa cut-off iCON™ concentrator (Pierce).

### Human brain tissue

Control and HD brain tissue were obtained from the Tissue Resource Center of the Alzheimer Disease Research Center (ADRC) at Massachusetts General Hospital. Post-mortem intervals were 4 hours.

### Immobilized metal affinity chromatography

In-vitro translated samples were diluted 1∶1 in binding buffer (20 mM sodium phosphate, pH 7.2, and 0.5 M NaCl) and applied to copper charged HiTrap Chelating Sepharose (Amersham) columns. All dilution, wash and elution solutions contained a protease inhibitor cocktail lacking EDTA and cysteine modifying inhibitors (Roche). Unbound protein was washed off using 5 mls of binding buffer. Bound proteins were then eluted using an imidazole gradient (8, 16, 32, 64, 128 mM; 1 ml for each fraction) stepwise in binding buffer. Columns were then stripped using 50 mM EDTA. Protein was precipitated from each fraction using 0.02% (w/v) deoxycholate, 10% (v/v) saturated trichloroacetic acid incubated overnight at 4°C, followed by centrifugation at 15000 g for 15 minutes. Pellets were then washed in cold acetone, dried, and then suspended in sample buffer. Samples were analyzed by Western blotting. For IMAC of brain supernatant, cortex was homogenized in 30 volumes of binding buffer containing protease inhibitor then 500 µl of supernatant fraction was applied directly to the column.

### Metal reduction assay

Copper and iron reduction assays were performed using a 96-well plate format essentially as described [13]. The final reaction contained 10 µM protein, 20 µM metal ion chloride and 360 µM bathocuproine or bathophenanthroline disulfonic acid. Protein concentrations were determined using the DC protein assay (Bio-Rad) using BSA as a standard.

### Huntingtin aggregation assay

Wild-type mouse cortices were homogenized in 30 volumes of TRIS pH 7.4 containing protease inhibitors lacking EDTA and N-ethyl maleimide (Roche) then samples clarified by centrifugation at 16000 g. One µg/µl protein with additives (see results) was incubated at 37°C then samples were resolved on a 5% denaturing polyacrylamide gel, transferred to PVDF then huntingtin detected by blotting.

### Mice husbandry

All protocols were conducted within NIH guidelines for animal research and were approved by the Institutional Animal Care and Use Committee. R6/2 mice express human huntingtin exon-1 containing a glutamine coding CAG. CAG140 knock-in mice have a CAG expansion within one of the endogenous full-length huntingtin alleles [21]. Both mouse lines were maintained on the B6/CBA crossed background. CAG expansion size ranges were 168–190 for R6/2 mice and 116–127 for knock-in mice.

### Copper and iron analyses

**Figure 1:**
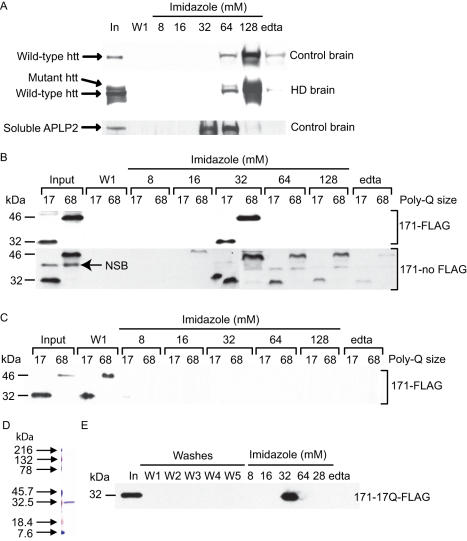
Full-length and N-terminal fragments of human huntingtin interact with copper (II) as measured by immobilized-metal affinity chromatography (IMAC). A. Full-length (FL) normal and mutant huntingtin from human motor cortex interact with copper and elute with peak centered at 128 mM imidazole. FL huntingtin has higher affinity for copper (II) than the copper-binding domain of human soluble APLP2. FL protein migrates at ∼350 kDa. B. N-171 fragments of human huntingtin with 17 or 68 glutamines interact with copper (II) and have identical elution profiles. Proteins with C-terminal FLAG tag elute as a single peak at 32 mM imidazole. Non-FLAG protein elutes primarily at 32 mM imidazole, but also at higher concentrations. NSB = non-specific band. C. N-171-flag fragments of wild-type and mutant huntingtin do not interact with iron (III). For iron-IMAC, iron (III) was loaded onto column at pH 3, adjusted to pH 7, then the IMAC procedure performed immediately. D. N-171-17Q fragment (wild-type) was expressed using the pGEX vector and purified (see methods). Coomassie gel analysis reveals high purity. E. Purified N-171-17Q-flag fragment of huntingtin interacts directly with copper (II) and elutes identically to the in-vitro transcription-translation expressed protein (Figure 1B).

Mice were deeply anesthetized with ketamine-xylazine then perfused transcardially for 3 minutes with 0.9% saline prepared using milli-Q water and containing 25 units/ml heparin. For figure 1E-F metals were measured by inductively-coupled plasma (ICP) spectroscopy. Brain samples were digested in 12 mls of nitric acid∶ perchloric acid in a 4∶1 (v/v) ratio, heated to 250°C on a hot plate until 1–2 mls of liquid remained. Samples were then cooled for 5 minutes then diluted to 12 mls using 0.5% nitric acid. Inductively-coupled-plasma (ICP) spectroscopy was performed as described [22]. All other metal quantifications in this study were made by ICP-mass spectroscopy, as described [23]. Buffers for the brain fractionation study were prepared using milli-Q water, treated with Chelex® 100 resin (Bio-Rad) to remove residual metals, then the pH adjusted to 7.2. Saline perfused frontal cortices were homogenized in five volumes of 10 mM TRIS then centrifuged at 20000 g for 30 minutes. The soluble fraction was removed and the pellet re-suspended in the same volume of 10 mM TRIS containing 1% Triton-X100 then centrifuged at 20000 g for 30 minutes to generate membrane and pellet fractions.

### SOD1 assay

Mice were perfused with saline as described above. Brain regions were homogenizing in 9 volumes of 10 mM HEPES (pH 7.4) containing a protease inhibitor with EDTA, sonicated briefly and then clarified. Activity was determined by measuring the inhibition of formation of a tetrazolium-formazan dye in the presence of superoxide (Dojindo Molecular Technologies) using bovine SOD1 as a standard.

### LDH and lactate assays

LDH activity was measured by determining the rate of formation of NAD+ at 340 nm in the presence of pyruvate. For lactate measurements brain regions were homogenized in 6% perchloric acid then clarified. The assay was performed in the presence of 50 mM imidazole (pH 7.5), 320 mM glycine, 320 mM hydrazine, 2.4 mM NAD+ and 5.5 u/ml of LDH. Lactate standards were used to calculate unknown values. *In-gel* detection of LDH activity was using a tetrazolium-salt-based chemistry (CytoTox 96®, Promega).

### Oxamate infusions

Eight-week-old wild-type mice were anesthetized with a tribromoethanol-based agent. Brain cannulas were installed at the following distances (in mm) from Bregma: 3 deep, 0.2 rostral and 1.9 left. Micro-osmotic pumps (Alzet®) were calibrated to delivery 5 nmoles/hour oxamate (pH 7.2). Glucose was used as an osmolarity control. Mice were sacrificed three days after surgery.

### Western blots

Brain regions were homogenized in 9 volumes of 50 mM Tris.HCl (pH 7.5), 1 mM EDTA and 1× HALT protease inhibitor (Pierce Biotechnology) using a Pellet pestel® (Kontes), sonicated briefly then centrifuged at 30000 g for 30 minutes at 4°C. Pellets were dissolved in 6 volumes of 50 mM Tris.HCl (pH 7.4), 150 mM NaCl, 0.5% deoxycholate (DOC), 1% SDS, 5 mM EDTA, 1 mM EGTA and HALT protease inhibitor by pipetting and brief sonication, then centrifuged at 16000 g for 15 minutes at 4°C. Primary antibody concentrations were all 1∶2000 except for anti-ATP7B (1∶600) and anti-β-actin (1∶5000). Secondary antibodies were all HRP-conjugated and detection used standard procedures.

### Quantitative PCR

Total RNA was extracted by homogenization of tissue in Trizol reagent (Invitrogen) using a Dounce homogenizer. After phase separation in chloroform, the supernatant fraction was applied directly to an RNAeasy (Qiagen) column and RNA purified with on-column DNAase 1 digestion. RNA integrity was confirmed using an Agilent 2100 Bioanalyzer (LabChip). Reverse transcriptase reactions used 0.5 and 0.8 µg RNA for striatum and cortex, respectively, and superscript II (Invitrogen). Quantitative PCR was essentially as described [*24*]. Primers used (upper/lower) were APP (5-gaagtcgccgaagaggaggaagtg/5-gttgtggtggtggtggcagtg), APLP1 (5-cgggagcagcagtgagga/5-ccggaaggagccagttcat), APLP2 (5-agcccatagtcagtctgtgttc/5-gcggtggaggtgagtgag). Amplicons were confirmed in size and sequence. Relative expression levels for each target were normalized to β-actin using the 2^−ΔΔT^ method [25].

### Statistical analysis

All data was analyzed using SAS software version 8.2 (SAS, Cary, NC) using the Students t-test or the generalized-linear-model procedure for ANOVA. The Dunn-Šidák method was used to correct for multiplicity. Mean±standard errors are presented.

## Results

### Copper interacts directly with N-terminal huntingtin

To address directly whether copper or iron can interact with huntingtin we used immobilized-metal-affinity chromatography (IMAC). Firstly, we performed IMAC experiments using cortical homogenate supernatant fractions from human control and HD brain. We found that both normal and mutant full-length huntingtin protein eluted from the copper column using 128 mM imidazole suggesting a fairly strong interaction with copper (Figure 1A). By comparison soluble amyloid precursor-like protein 2, a protein with a known copper binding site [26] eluted at 32–64 mM (Figure 1A) while prion protein (PrP) is known to elute at 150 mM imidazole [27]. To determine whether polyglutamine containing N-terminal huntingtin fragments can interact with copper, we expressed N-terminal fragments of wild-type and mutant huntingtin by *in-vitro* transcription-translation (IVTT) then performed IMAC experiments. Because we were unable to express mutant exon-1 huntingtin by IVTT we focused on a longer fragment of N-terminal huntingtin containing the first 171 amino acids of huntingtin with 17 or 68 glutamines (17Q/68Q), for wild-type and mutant protein respectively. We used constructs expressing a C-terminal FLAG tag for most of our experiments. N171-17Q/68Q FLAG fragments both eluted at 32 mM imidazole (Figure 1B). Experiments using N171 fragments lacking FLAG revealed that N171 also eluted at 32 mM imidazole, but continued to elute at 64 and 128 mM imidazole indicating that FLAG attenuates N171-copper interaction (Figure 1B). Further, the N-171 fragments failed to bind to iron (III) loaded IMAC columns (Figure 1C). To determine if the N-171 interaction with copper is direct or depends on another protein we generated purified N171 using the pGEX bacterial expression system (Amersham). N171-17Q was pure as measured by Coomassie staining (Figure 1D). Further, this fragment eluted identically to the IVTT expressed fragment (Figure 1B and E) indicating that copper (II) interacts directly with N-171 huntingtin. Bacterially generated N-171-68Q aggregated on the copper (II)-IMAC column, however, it partially eluted predominantly in the 32 mM imidazole peak (not shown).

### Copper-huntingtin interaction involves histidine residues

**Figure 2:**
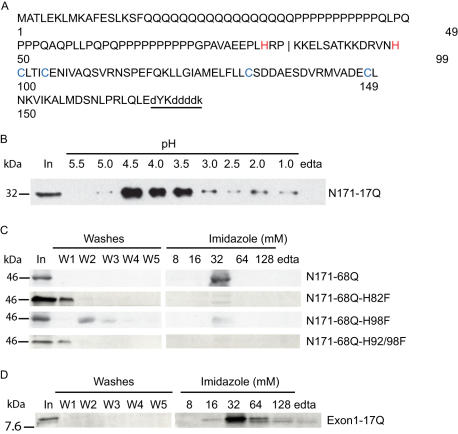
Interaction of N-171 huntingtin with copper (II) by IMAC involves histidine 82 and 98. A. N171-17Q huntingtin contains several potential copper (II) coordinating residues, two histidines (red) and four cysteines (blue). The vertical line represents the exon-1-2 boundary. Flag tag sequence (underlined) partially overlaps with huntingtin sequence (upper case). B. Purified N-171-17Q huntingtin fragment elutes from copper column at pH 4.5–3.5 consistent with interaction with histidine residue(s). Elution solutions were prepared in 20 mM citrate-buffer. C. Modification of histidine 82 and/or 98 to phenylalanine results in elution of protein in washes indicating that both histidines are necessary for interaction with copper (II). D. Huntingtin exon-1-17Q fragment is sufficient for copper (II) interaction.

N171 huntingtin protein contains several candidate copper (II) coordinating residues (Figure 2A). To characterize the nature of an N-171 huntingtin-copper interaction we determined the pH dependency of the N171-17Q-copper interaction. Purified N-171-17Q eluted predominantly in the pH range 4.5–3.5 consistent with a histidine interaction (Figure 2B). Modification of cysteine residues on purified N-171-17Q using N-ethyl-maleimide failed to affect binding (not shown). Mutation of histidines 82 and 98 to phenylalanine in N-171-68Q protein both resulted in loss of binding (Figure 2C) indicating that in N171 both residues are essential for an interaction with copper. However, human exon-1 (17Q) huntingtin (N84), which lacks histidine 98, interacted with copper (II) and eluted at 32 mM imidazole to EDTA (Figure 2D) suggesting it has a different conformation and mode of interaction with copper than N171.

### Copper(II) is reduced by and promotes aggregation of huntingtin

**Figure 3:**
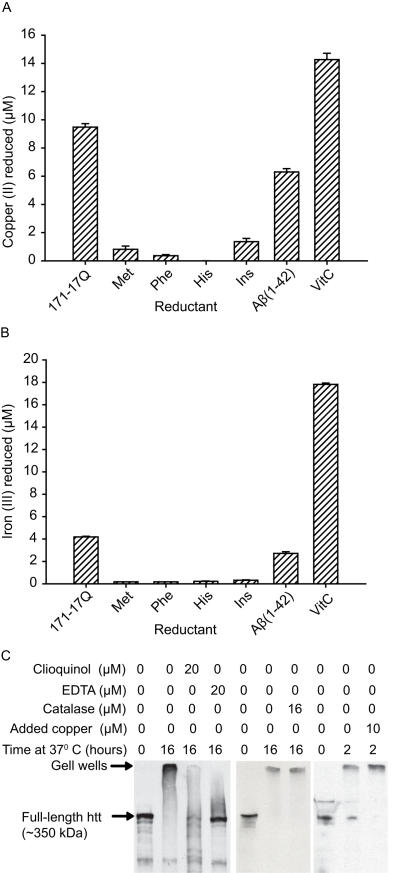
Copper (II) is reduced by N171-17Q huntingtin and promotes aggregation of cell-free full-length mouse huntingtin. A. N-terminal huntingtin (N-171-17Q) reduces copper (II) as measured by the bathocuproine assay. B. Iron (III) is reduced by N171-17Q to a lesser extent than copper as measured by bathophenanthroline assay. Met = methionine, Phe = phenylalanine, His = histidine, Ins = insulin, VitC = ascorbate. Purified N171-17Q proteins were incubated for 1 hour at 37°C in the presence of 20 µM of copper (II) or iron (III) and 360 µM bathocuproine or bathophenanthroline for copper (II) and iron (III) reduction assays, respectively. n = 4. C. Full-length wild-type mouse huntingtin aggregates following incubation at 37°C. Aggregation is promoted by 10 µM copper (II) and inhibited by EDTA and the brain permeable chelator, clioquinol. Aggregation is not inhibited by co-incubation with catalase.

To determine if the copper-N171 complex is redox-active we determined whether N171 could reduce copper (II) to copper (I). The experiment was performed using the 17Q protein only because we were unable to sufficiently purify mutant N171. N171-17Q reduced copper (II) at a 1∶1 stoichiometric ratio (protein∶Cu^2+^) within 1 hour (Figure 3A). N171-17Q also reduced iron (III), but to a lesser extent than copper: approximately 1 mole of Fe^3+^ was reduced per 2 moles of protein (Figure 3B). We found that wild-type full-length huntingtin from mouse brain supernatant fractions form SDS and β-mercaptoethanol resistant aggregates upon incubation at 37°C (Figure 3C). Because wild-type huntingtin fragments can aggregate *in-vivo* [28] and because polyglutamine length within the normal range modifies cell phenotype [5] we used wild-type huntingtin to investigate the effect of chelators and copper on aggregation. We found that the brain permeable metal chelator clioquinol and EDTA inhibited formation of aggregates. Aggregation was not blocked by catalase (indicating that it is not mediated by H_2_O_2_), but was promoted by copper (Figure 3C).

### Copper and iron are increased in mouse HD brain

**Figure 4:**
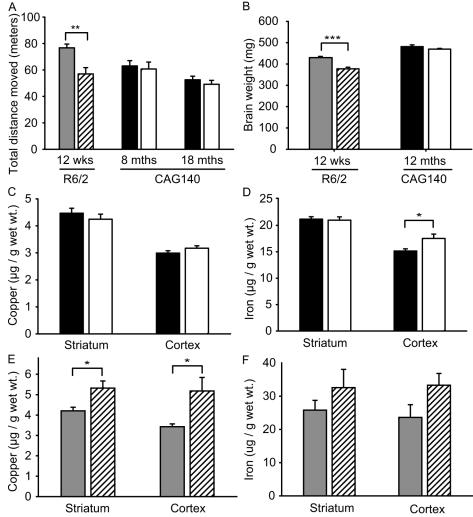
Brain copper and iron levels in pre-clinical and impaired Huntington's disease (HD) mice. A–B. Twelve-week-old R6/2 and 12-month-old CAG140 mice represent late stage and pre-clinical HD, respectively. A. Open-field activity of R6/2 transgenic and CAG140 knock-in mice. R6/2 mice at 12-weeks have significantly decreased activity. CAG140 mice at 8–18 months are the same as wild-type littermates consistent with pre-clinical disease. B. Brain weights of 12-week-old R6/2 mice are decreased 12% consistent with severe brain atrophy and late-stage HD. Twelve-month-old CAG140 mouse brain weights are normal (p = 0.2367). C–D. Copper and iron levels in brains of 12-month-old CAG140 HD knock-in mice (pre-clinical HD) as measured by inductively-coupled-plasma (ICP) mass spectroscopy. C. Copper levels are unaltered. D. Iron levels are increased 15% in cortex. E–F. Copper and iron levels in brains of 12-week-old R6/2 HD mice (late-stage HD) as measured by ICP spectroscopy. E. Copper levels are significantly increased in striatum and cortex. F. Striatal and cortical iron levels are increased, but not significantly (p-values are 0.2897 and 0.0782, respectively). n = 10–14. Values are shown as mean±SEM. Bars: white = CAG140; cross-hatched = R6/2; gray = wild-type litter mates of R6/2; black = wild-type litter mates of CAG140. p-values: *<0.05, **<0.01, *** = p<0.0001

Copper and mutant huntingtin accumulation in HD could increase the probability of an interaction occurring in brain. To determine if redox-active metal levels increase with HD progression we measured copper and iron concentrations in striata and frontal cortices of 12-month-old knock-in CAG140 and 12-week-old R6/2 transgenic HD mice. CAG140 mice at 8–18 months had no deficits in open-field activity (Figure 4A) and brain weights were normal at 12-months (Figure 4B), consistent with pre-clinical HD. R6/2 mice had open-field deficits (Figure 4A) and a 12% reduction in brain weight (Figure 4B) consistent with advanced HD. Despite the absence of deficits in our CAG140 mice cortical iron levels were significantly increased 15% (Figure 4D). In R6/2 mice with advanced HD copper was significantly increased 26% and 51% in striatum and cortex respectively (Figure 4E). Iron levels were increased in both regions in the R6/2 brains (Figure 4F), although not reaching statistical significance in this experiment (see below).

**Figure 5:**
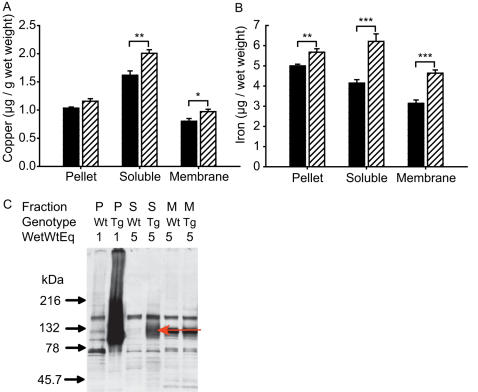
Mutant huntingtin, copper and iron distribution in biochemical fractions of 12-week-old HD mouse cerebral cortex. A. Copper is increased in soluble and membrane fractions. B. Iron is increased in soluble, membrane and pellet fractions. Metals were measured by ICP-MS. n = 10–11. p-values after Dunn-Šidák correction for multiple testing *<0.05, **<0.01, ***<0.001 C. Distribution of mutant huntingtin in pellet (P), soluble (S) and membrane (M) fractions as determined by Western blotting using a polyglutamine specific antibody (IC2-Chemicon). Pellets were treated with 90% formic acid for 1 hour at 37°C, lyophilized then resuspended in SDS-PAGE buffer. Mutant huntingtin is identified by the presence of a band in transgenic mice (Tg) but not wild-type (Wt) mice (red arrow for soluble fraction). WetWtEq = wet weight equivalents.

To identify *in-vivo* evidence of a metal N-terminal huntingtin interaction we determined if mutant huntingtin distribution co-localizes with increases in metal levels in brains of 12-week-old R6/2 HD mice. We measured cortical metal levels by ICP-MS in three biochemical fractions and correlated these with mutant huntingtin protein levels in the same fractions. Metal and huntingtin measurements were all normalized to wet weight to allow the distribution of metals and mutant huntingtin to be compared quantitatively. After correction for multiple testing, significant increases in copper were present in soluble and membrane fractions (Figure 5A). Iron levels showed greater increases in these regions and were also significantly increased in the pellet fraction (Figure 5B). Zinc concentrations were unaltered in all fractions (not shown). As expected, we found that R6/2 brain contains a large excess of mutant huntingtin in insoluble compared to soluble forms (Figure 5C). Mutant huntingtin was not detected in the membrane fraction. Neither pellet nor soluble mutant huntingtin could be detected by Coomassie staining in parallel gels (not shown). Based on the detection limit of Coomassie blue (∼100 ng) we estimated that at 12-weeks≤84 nmoles mutant huntingtin/g wet weight is present in R6/2 HD cortex. From this we calculated that in the pellet fraction there is still at least a 17-fold molar increase of copper over mutant huntingtin. Therefore, the increase in copper in R6/2 cortex is sufficiently high to be compatible with binding to all the mutant huntingtin. However, as the increase in copper is many folds greater than the molar concentration of mutant huntingtin, significant amounts of excess copper are available to interact at non-huntingtin sites.

### Lactate dehydrogenase activity is decreased in mouse HD brain and inhibition is sufficient to cause neurodegeneration

**Figure 6:**
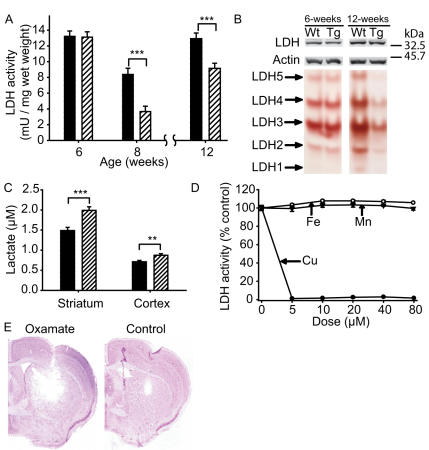
Decreased L-lactate dehydrogenase (LDH) activity in R6/2 HD mice is relevant to neurodegeneration. For A and C, black = wild-type, cross-hatched = transgenic. A. Activity of the copper-sensitive enzyme LDH is decreased in forebrains of R6/2 HD mice from 8-weeks. Time points were determined on consecutive generations of mice, thus only within time point comparisons are valid. n = 10. B. Total actin-normalized LDH monomer levels are unaltered (n = 4) but all five LDH isoenzyme activities are decreased at 12-weeks (n = 4, p = 0.0421). C. Lactate levels are increased in striatum and cortex of 12-week-old R6/2 HD mice. D. LDH is exceptionally sensitive to copper, but not iron or manganese, mediated inactivation. Five µg/ml purified LDH (Roche) was incubated with copper (II), iron (III), manganese (II) chloride in chelex-treated PBS for 1 hour at 37°C before LDH analysis. n = 8 Symbols: black circles = copper, white circles = iron, triangles = manganese. P-values: *** = p<0.0001, ** = p<0.001, E. The LDH inhibitor oxamate results in acute degeneration in wild-type mice when delivered intra-striatially, as compared to control treated mice.

Our data so far suggested that not all the increased copper (and iron) are in the same biochemical fraction as mutant huntingtin. We reasoned that this excess copper could be in a toxic form unassociated with mutant huntingtin. Firstly, we sought evidence that increased copper could be part of a protective response. As the R6/2 mouse model of HD mounts a brain anti-oxidant response to mutant huntingtin [24] we first determined whether this response includes upregulation of the cuproenzyme SOD1. However, there was no significant increase in SOD1 activity or protein levels in striatum or cortex of 12-week R6/2 mice (Figure S1). To look for evidence of copper toxicity we determined if there was a reduction in activity of the copper-sensitive enzyme LDH [15]. LDH activities were decreased in forebrains of affected HD mice from 8-weeks (Figure 6A). Further, there were no significant changes in actin-normalized expression levels of LDH monomer protein as measured by Western blotting (Figure 6B) indicating that reduced LDH activity is not the result of decreased protein levels in the R6/2 mouse. All five LDH isoenzymes had decreased activities in 12-week R6/2 HD mice (Figure 6B). Averaging across the five LDH isoenzymes there was a 55% decrease in actin-normalized activity (p = 0.0421) which is comparable to the enzymatic activity data. Further, at the same age we found increased L-lactate levels in R6/2 striatum and cortex (Figure 6C). We investigated the sensitivity of LDH to copper and other metals. LDH was inhibited>95% after 1 hour incubation in 5 μM copper (II) but there was no effect of manganese (II) or iron (III) at up to 80 µM (Figure 6D). These findings are consistent with copper inhibiting LDH in brain, resulting in altered brain L-lactate metabolism. To determine whether LDH inhibition in brain can contribute to neurodegeneration we infused the LDH inhibitor, oxamate, into the striatum of wild-type mice. After three days there was significant striatal degeneration in oxamate treated, but not control mice (Figure 6E).

### Decreased expression of the copper export protein APP in HD brain

**Figure 7:**
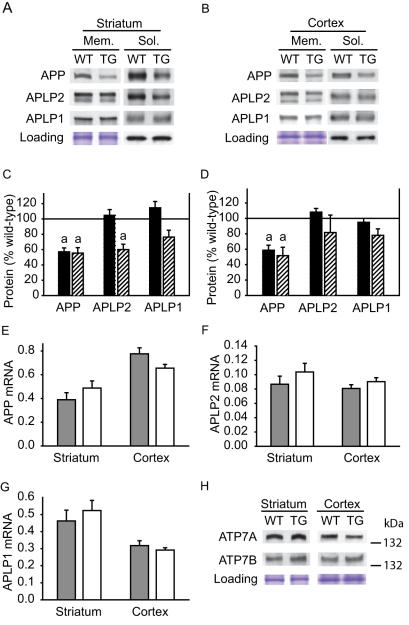
Amyloid precursor protein (APP), a copper exporting protein, is decreased at the protein but not transcript level in the brains of 12-week-old HD transgenic mice. A–D. Soluble and membrane forms of APP are significantly decreased in striatum and cortex of R6/2 HD transgenic mice. APP-like protein 2 (APLP2) is decreased in striatal soluble fraction only. APLP1 levels are unaffected. β-actin is loading control for soluble fraction. C–D. Expression levels for transgenic mice are shown relative to respective wild-types (normalized to 100%). Black bars = membrane fraction, cross-hatched bars = soluble fraction. n = 11, a = p<0.0001 E–G. Transcripts coding for APP, APLP2 and APLP1 are unaltered in striatum and cortex. n = 10 Gray bars = wild-type, white = R6/2 transgenic. H. Analysis of copper-transporting ATPase expression in HD mice. Menke's-disease protein (ATP7A) expression is unaltered. Wilson's disease protein (ATP7B) is increased in cortex only (p = 0.0174). n = 7–10, WT = wild-type, TG = transgenic.

To characterize further the state of copper homeostasis in R6/2 mice we analyzed the expression of several copper homeostatic proteins. We analyzed amyloid precursor protein (APP) and APP-like protein 2 (APLP2) because mice lacking these proteins have increased brain and liver copper levels [29]. Because APP family proteins are cleaved within their single transmembrane region to yield an N-terminal extracellular soluble form we measured APP and APLP2 protein levels in soluble and membrane fractions. Both forms of APP were highly significantly decreased (∼45%) in striatum and cortex of 12-week-old R6/2 mice (Figure 7A–D). APLP2 was decreased in the soluble fraction of striatum only and APLP1 levels were unaltered. APP, APLP2 and APLP1 mRNA transcript levels were unaltered (Figure 7E–G). The copper-transporting ATPase, Wilson's disease protein (ATP7B) was increased 40% in cortex, but not in striatum (Figure 7H). There were no significant changes in copper-transporting Menke's disease protein (ATP7A) expression (Figure 7H).

## Discussion

Accumulation of basal ganglia copper or iron results in neurodegeneration [30], [31]. In this study, we tested the hypothesis that in HD brain copper accumulates and promotes disease progression by pro-oxidant protein interactions. We focused on two proteins: huntingtin, because of its centrality to HD [32], and lactate dehydrogenase (LDH) because of its documented sensitivity to copper [15], necessity for normoxic brain energy metabolism [18] and evidence for altered lactate metabolism in HD brain [19]. We tested this hypothesis in experiments using post-mortem brain tissue from HD patients, human huntingtin fragments expressed biochemically and in transgenic mice, and using a knock-in model of HD expressing full-length mutant huntingtin. Our findings support the role of excess copper as a potentiating factor in HD.

We used immobilized metal affinity chromatography (IMAC) to investigate whether huntingtin interacts with copper. IMAC has previously been used to investigate copper-protein interactions involved in neurodegenerative disease [26]. Experiments revealed that N171 containing a normal (17Q) or expanded (68Q) glutamine tract interacted with copper (II), but not iron (III) or zinc (not shown). We identified histidines 82 and 98 as both essential for copper interaction (Figure 2B–C) suggesting that they coordinate a single copper ion. The exon-1-17Q fragment (N84) also interacted with copper (II) (Figure 2D) indicating that our findings of a copper-N-terminal huntingtin interaction are relevant to the neurodegenerative phenotype of the R6/2 HD mouse which expresses exon-1 of mutant huntingtin. However, as the N171 fragment with the H98F mutation did not bind to the IMAC column it suggests that exon-1 and N171 fragments coordinate copper differently (see Figure 2A). We have no direct evidence that glutamine expansion affects copper-binding. However, sequences flanking the polyglutamine tract have profound influences on huntingtin toxicity [33], therefore, our findings are consistent with a process that could modulate huntingtin toxicity in-vivo.

We found that N171-17Q protein is a strong reducer of copper (II) indicating that copper (II) and N171-17Q huntingtin not only interact in solution but that an interaction can result in electron transfer. The overall level of copper reduction by N171-17Q was much greater than insulin, a protein oxidized by copper [34], and similar to that reported for Abeta(1–42) [13] indicating that N171 protein is very redox active. Iron (III) reduction by N171-17Q did occur, but was much less than copper (II) (Figure 3A–B). This suggests that iron may interact with N-terminal huntingtin in solution even though an interaction was not detected by IMAC. Mutant huntingtin aggregates have recently been shown to be centers of reactive oxygen species (ROS) production in cultured cells [35]. The authors implicated iron in this process because ROS production was inhibited when the metal chelator deferoxamine was added to cell culture medium, even though deferoxamine is not specific for iron. Despite this interpretation, these results are in general agreement with each other indicating that N-terminal huntingtin redox activity may be important in HD pathogenesis.

Insoluble huntingtin aggregates are one pathologic hallmark of HD [2]. Our finding that copper promotes and metal chelation inhibits cell-free formation of SDS and β-mercaptoethanol resistant huntingtin aggregates (Figure 3C) suggests that copper could modulate mutant huntingtin aggregation in-vivo. These findings raise the possibility that metal chelators could be neuroprotective in HD by inhibiting early structural changes and silencing redox activity of mutant huntingtin. The brain permeable copper and iron chelator clioquinol is neuroprotective and decreases brain aggregate load in R6/2 HD mice [36]. Further, epigallocatechin-gallate, a green tea flavonoid and copper chelator [37] modulates early events in huntingtin misfolding and reduces toxicity in a Drosophila model of HD [38]. While the role of microscopically visible aggregates in HD pathogenesis is controversial, our findings indicate that copper interacts with mutant N-terminal huntingtin monomers (Figure 1). This indicates that copper may modulate aggregation by influencing proximal mutant huntingtin structural changes in the cascade from monomer to oligomer and microscopic aggregate. The exact mechanism by which this occurs is unclear. Possibilities include copper-mediated conformational change, huntingtin oxidation, and proteolysis.

Copper and iron levels were significantly increased in brains of 12-week R6/2 HD mice, corresponding to late-stage HD. However, only iron was increased in the brains of 12-month CAG140 HD mice, corresponding to pre-clinical HD (Figures 4A–D). The effects in R6/2 mice are unlikely to be due to shifts in cell populations with advancing HD because cortical zinc concentrations were unaltered (not shown). Our findings demonstrate that these models may be useful for understanding the altered copper and iron homeostasis that occurs in human HD [6]. They also argue for an early role of iron in HD. Further, because we did not detect increases in copper in CAG140 mice with pre-clinical disease our results are most consistent with copper potentiating HD progression, rather than acting very early in disease pathogenesis. Greater involvement of cortex for iron (CAG140 mice) and copper (R6/2 mice), rather than striatum, is consistent with HD mechanisms in which cortical dysfunction results in striatal degeneration [39], [40].

To address whether the distributions of increased copper and mutant huntingtin in different biochemical fractions are compatible with an interaction occurring in brain we correlated their distributions in R6/2 cortex. We also measured iron because an interaction with aggregated huntingtin cannot be excluded based on our biochemical findings. We found significant increases in copper and iron in specific fractions (Figure 4A–B). The molar increase in copper and iron was several times greater than the amount of mutant exon-1 huntingtin present (see results). Therefore, our data suggest that not all the increased copper can be bound to mutant huntingtin if there is a 1∶1 binding stoichiometry and that some excess metals are likely at other cellular or sub-cellular sites where they may be mediating oxidative injury.

Copper inactivates LDH by direct oxidation of an active-site cysteine residue [15]. In our studies, forebrains of 12-week-old R6/2 mice had reduced LDH activities and increased L-lactate levels suggesting that disturbances in lactate metabolism could be directly due to reduced LDH activity. It has recently been shown that LDH-B isoform mRNA levels are decreased in N171-82Q mice and human HD brain due to decreased signaling through the PPARγ coactivator 1α (PGC-1α) pathway [20]. Further, N171-82Q mice have decreased ability to utilize lactate as an energy substrate [20]. While defects in PGC-1α signaling are also present in R6/2 HD mice [41] we did not detect decreases in total LDH monomer protein levels as measured by Western blotting (Figure 6B). Despite unaltered LDH monomer levels, both our biochemical and in-gel LDH activity assays demonstrate an age-dependent decline of LDH activity in R6/2 HD brain (Figure 6A-B). Given the increased forebrain copper in R6/2 mice at 12-weeks (Figure 4E) and the sensitivity of LDH to copper-mediated inactivation (Figure 6D) we suggest that reduced LDH activity is, at least in part, the result of copper-mediated enzymatic inhibition. This interpretation is supported by our fractionation studies suggesting that increased copper is not entirely associated with mutant huntingtin. The LDH inhibitor oxamate resulted in striatal degeneration (Figure 6E) indicating that a defect in LDH activity is sufficient to contribute to neurodegeneration in HD mice. Taken together, these data suggest that copper interferes with lactate metabolism in HD brain by inhibiting LDH.

Copper levels could increase in HD brain for a number of reasons including increased expression of neuroprotective cupro-proteins, altered expression of copper-homeostatic proteins and accumulation of mutant huntingtin containing a copper-binding site. Liver copper (and iron) levels were unaltered in R6/2 mice at 12-weeks ruling out systemic accumulation as a factor (not shown). R6/2 HD mice mount a compensatory anti-oxidant response [24]. Further, SOD1 is an important anti-oxidant and represents about 1% of total brain protein; however, we did not detect significant shifts in protein or activity levels that might explain increased copper levels in R6/2 mice (Figure S1). We analyzed levels of several proteins implicated in copper homeostasis. APP and APLP2 knock-out mice have increased cortical copper concentrations [29]. We found that R6/2 HD mice had decreased levels of APP and APLP2 (Figure 7A-D) suggesting that this could be one factor explaining increased copper. Similar to R6/2 mice, APP knock-out mice have decreased grip strength and locomotor activity [42]. APP and APLP2 transcript levels were unaltered in R6/2 mice and we did not detect degradation products by Western blotting suggesting that decreased levels of these proteins may result from translational inhibition. In addition to its role in copper-export APP has neuroprotective [43] and neurotrophic effects [44], [45]. Therefore, decreased APP levels in R6/2 HD mice could contribute to neurodegeneration by copper-dependent and independent mechanisms.

We have provided evidence that copper interactions with mutant huntingtin and LDH are important in HD pathogenesis. While the interaction of N-terminal huntingtin with copper is a new finding, redox-mediated mechanisms of mutant huntingtin toxicity are compatible with established mechanisms of huntingtin toxicity such as dysregulation of axonal transport and transcription. In addition to the mechanisms proposed, copper could promote HD progression in additional ways. For example, in the presence of hydrogen peroxide, ions of copper are highly reactive towards DNA, several fold more reactive than iron [46], [47] and there is evidence for abundant DNA oxidation in HD brain [4], [48]. Further, the neurotoxic metabolite 3-hydroxykynurenine is increased in HD [49], [50] and in-vitro reacts more readily with copper than iron to generate hydrogen peroxide [51]. Despite the proposed importance of copper, iron may play an important and early role in HD, and this requires more investigation. More work is also needed to further quantify affinities and specificities of metal ion binding to N-terminal huntingtin fragments and to better understand the redox chemistry involved. When taken together however, our findings underscore the relevance of metal-protein chemistries in HD brain and suggest neuroprotective stratagies.

## Supporting Information

Figure S1 SOD1 protein and activity levels in striatum and cortex of HD mice. A. SOD1 protein levels are unaltered in cortex and striatum of HD mice. B. SOD1 activity is unaltered in striatum and non-significantly increased in cortex (p = 0.0962). All measurements were at 12-weeks of age. n = 10, black bars = wild-type, cross-hatched = HD transgenic.
(0.62 MB EPS)
